# Piceatannol Alleviates *Clostridium perfringens* Virulence by Inhibiting Perfringolysin O

**DOI:** 10.3390/molecules27165145

**Published:** 2022-08-12

**Authors:** Guizhen Wang, Hongtao Liu, Yawen Gao, Xiaodi Niu, Xuming Deng, Jianfeng Wang, Haihua Feng, Zhimin Guo, Jiazhang Qiu

**Affiliations:** 1 Key Laboratory of Zoonosis Research, Ministry of Education, College of Veterinary Medicine, Jilin University, Changchun 130062, China; 2 Measurement Biotechnique Research Center, College of Biological and Food Engineering, Jilin Engineering Normal University, Changchun 130052, China; 3 College of Food Science and Engineering, Jilin University, Changchun 130062, China; 4 Department of Clinical Laboratory, The First Hospital, Jilin University, Changchun 130021, China

**Keywords:** piceatannol, perfringolysin O, *Clostridium perfringens*

## Abstract

*Clostridium perfringens* (*C. perfringens*) is an important foodborne pathogen that can cause diseases such as gas gangrene and necrotizing enteritis in a variety of economic animals, seriously affecting public health and the economic benefits and healthy development of the livestock and poultry breeding industry. Perfringolysin O (PFO) is an important virulence factor of *C. perfringens* and plays critical roles in necrotic enteritis and gas gangrene, rendering it an ideal target for developing new drugs against infections caused by this pathogen. In this study, based on biological activity inhibition assays, oligomerization tests and computational biology assays, we found that the foodborne natural component piceatannol reduced pore-forming activity with an inhibitory ratio of 83.84% in the concentration of 16 µg/mL (IC_50_ = 7.83 µg/mL) by binding with PFO directly and changing some of its secondary structures, including 3-Helix, A-helix, bend, and in turn, ultimately affecting oligomer formation. Furthermore, we confirmed that piceatannol protected human intestinal epithelial cells from the damage induced by PFO with LDH release reduced by 38.44% at 16 µg/mL, based on a cytotoxicity test. By performing an animal experiment, we found the *C. perfringens* clones showed an approximate 10-fold reduction in infected mice. These results suggest that piceatannol may be a candidate for anti-*C. perfringens* drug development.

## 1. Introduction

*Clostridium perfringens* (*C. perfringens*), a ubiquitous Gram-positive anaerobic bacterium, causes a variety of diseases in humans and animals ranging from necrotizing enteritis to intestinal toxemia and gas gangrene [1,2]. Necrotizing enteritis has a higher incidence, and its mortality rate can reach as high as 50% in extremely serious cases [3]. Approximately 37% of commercial broilers worldwide are affected by this disease, and USD 50–60 billion in economic losses are generated from necrotizing enteritis each year [4], seriously affecting the development of livestock and poultry breeding. Gas gangrene is another clinically fatal disease caused by *C. perfringens*, which is mainly characterized by muscle tissue necrosis and rapid progression (known as muscle necrosis) [5]. This disease leads to systemic blood poisoning, shock and even death if treatment is delayed [5,6,7]. The mortality of gangrene is high, especially in periods of war and natural hazards such as earthquakes, and can reach 50–80% [8], posing a serious threat to human life and health.

*C. perfringens* secretes multiple exotoxins to promote successful infection [9], among them, perfringolysin O (PFO) has been demonstrated to be responsible for the development of necrotizing enteritis and gas gangrene [10]. PFO is encoded by the *pfoA* gene and comprises 499 residues with a molecular weight of approximately 54 kD [10]. As a member of the cholesterol-dependent cytolysin toxin family, PFO is secreted as a soluble monomer that binds with cholesterol on the mammalian cell membrane and forms oligomers consisting of 40–50 monomers through spatial conformational changes to penetrate the cell membrane, leading to large pores with a diameter of 300–450 Å [11], triggering cytotoxicity and inflammatory reactions, and ultimately interfering with the normal physiological function of cells. Almost all *C. perfringens* strains secrete PFO [12]. By lysing intestinal endothelial cells, PFO leads to capillary bleeding and causes necrotizing enteritis, which is accompanied by sudden death. Pathological damage and bleeding lesions were observed when infected animals were dissected. The symptoms of necrotizing enteritis, along with pathological damage and bleeding lesions in the intestine, were significantly alleviated when animals were infected with a *C. perfringens* strain lacking the *pfoA* gene [12]. PFO helps *C. perfringens* escape from the phagocytic vesicles of macrophages to avoid the scavenging effect of the immune system in the early stage of infection and promotes the survival of *C. perfringens* in the host [13]. PFO promotes hematoma and endothelial cells to secrete adhesion factors and chemokines that may trigger platelets to secrete activation factors. Activation factors cause platelets and hematoma to adhere to vascular endothelial cells and form thrombi, which reduce the blood flow rate, result in hypoxia in the infected tissues and finally cause gas gangrene development. The pathologic injury severity of gas gangrene induced by the *pfoA* mutant was significantly reduced [12,14,15]. The critical role of PFO in the development of gas gangrene and necrotizing enteritis induced by *C. perfringens* renders it an important target for the development of new anti-infective drugs.

Piceatannol, a foodborne natural compound, is widely found in various fruits, vegetables and medicinal plants. Piceatannol has been reported to have a variety of biological and pharmacological functions, such as antioxidant, antiviral, antiatherosclerotic, anticancer and antiparasitic effects [16,17,18,19]. In this study, we found that piceatannol can inhibit the pore-forming activity of PFO through a direct binding, which changes the spatial conformation of PFO, inhibiting the formation of its oligomers and ultimately resulting in a decline in pore-forming activity. Furthermore, piceatannol alleviated the cytotoxicity mediated by PFO and reduced the colonization of *C. perfringens* in the muscle tissues of infected mice.

## 2. Materials and Methods

### 2.1. Bacterial Strain and Reagents

*C. perfringens* was purchased from American Type Culture Collection (ATCC13124) and was stationarily cultured in brain heart extract broth (BHI) in an anaerobic environment at 37 °C. Cytotoxicity kits were purchased from Roche (Basel, Switzerland). Dimethyl sulfoxide (DMSO) was purchased from Sigma-Aldrich (St. Louis, MO, USA). Dulbecco’s modified Eagle’s medium (DMEM) and fetal calf serum were purchased from HyClone (Logan, UT, USA). Sterile defibrinated rabbit blood was purchased from Zhengzhou Jiulong Biological Products Co., Ltd. (Zhengzhou, China). Rabbit-sourced PFO antibody was acquired from Cusabio Biotech Co., Ltd. (Wuhan, China). BHI, anaerobic culture bags and carbon dioxide production packages were purchased from Qingdao Hope Bio-Technology Co., Ltd. (Qingdao, China). Piceatannol with purity greater than 98% was purchased from Chengdu Ruifensi Biological Technology Co., Ltd. (Chengdu, China).

### 2.2. Pore-Forming Activity Inhibition Assays

PFO (1 µg) was incubated with various concentrations of piceatannol (0, 4, 8, 16 µg/mL) in 500 µL phosphate buffer saline (PBS) buffer at 37 °C for 20 min, followed by the addition of sterile defibrinated rabbit blood at a final concentration of 2.5% and co-cultured for 10 min under the same conditions. The supernatant of each sample was used to detect the optical density at 543 nm (OD_543_) [20,21]. Sterile defibrinated rabbit blood in sterile water or sterile PBS was used as a positive or negative control, respectively. The inhibitory ratio was calculated based on the equation of (1−OD_543treatment_/OD_543untreatment_) × 100.

### 2.3. Molecular Docking vs. PFO

Piceatannol, which was used as the ligand, was obtained from PubChem, and the topological structure was generated by using AmberTools [22,23,24]. The crystal structure of PFO was obtained from the Protein Data Bank (PDB) with PDB code 1PFO and treated with AutoDockTools 1.5.6 [25]. AutodockVina [26] was used to perform the docking calculation by using a semiflexible docking method. Gromacs 2020.6 [27] was used to carry out molecular dynamics simulation assays, and the Amber99SB [28] force field and the TIP3P water model were used for this work. The final simulation was arranged after energy minimization, temperature and pressure equilibrium. Root mean square deviation (RMSD) values were monitored for both the receptor and the ligand to evaluate the conformational change during the binding process. The interaction energy was analyzed based on the molecular mechanics Poisson–Boltzmann surface area (MM-PBSA) method [29], hydrogen bonds (H-bonds) were analyzed during binding, and the secondary structures were analyzed based on a dictionary of protein secondary structures [30,31]. Graphical visualization was performed using UCSF Chimaera 1.16 [32]. Other details were based on previously described methods [33].

### 2.4. Oligomer Formation Inhibition Assays

PFO (0.5 µg) was preincubated with various concentrations of piceatannol (0, 32, 128 µg/mL) in 80 µL PBS buffer at 37 °C for 20 min, and then sterile defibrinated rabbit blood was added, followed by cooling on ice for 2 min to induce oligomer formation. Then, 5× SDS loading buffer without 2-ME was added to each sample, and the samples were treated at 55 °C for 10 min. Each sample was separated by a 6% separation gel, and the proteins were transferred onto a PVDF membrane. The proteins were detected by the PFO antibody (1:1000) and secondary antibodies (1:10,000) after sealing with 5% skim milk powder solution. Images were acquired from a gel imaging system.

### 2.5. Cytotoxicity Assays

Human colorectal cancer epithelial cells (Caco-2) were seeded onto 96-well plates (3 × 10^4^ cells/well) and cultured overnight at 37 °C under 5% CO_2_. The next day, the cell culture medium was replaced with serum-free DMEM, and 5 µg PFO protein and different concentrations of piceatannol (0, 4, 16 µg/mL) were added into each well for coculture under anaerobic conditions. Four hours later, the plate was centrifuged at 1000 rpm for 10 min, and 100 µL of the supernatant was used to detect lactate dehydrogenase (LDH) release using a kit. LDH release from cells treated with 1% Triton-X 100 or DMEM only was used as a positive or negative control, respectively.

### 2.6. Animal Infection

The animals used in this study were female BALB/c mice aged 6–8 weeks purchased from Liaoning Changsheng Biotechnology Co., Ltd. (Liaoning, China). Three groups (blank group, infection group, treatment group) were established randomly. *C. perfringens* ATCC13124, which produces PFO, was intramuscularly injected (5 × 10^7^ CFUs/mouse) into the infection and treatment group mice after culture and harvest. Twenty microliters of piceatannol dissolved in DMSO at a final concentration of 50 mg/mL was subcutaneously injected into each mouse in the treatment group 2 h after infection, and an equal volume of the solvent was injected into the infection group mice. The mice in each group were euthanized with anesthesia after 48 h, and the leg muscle was obtained and homogenized in sterile PBS. Then, the homogenate was coated onto BHI agar plates and cultured overnight under anaerobic conditions, and clones were statistically analyzed.

### 2.7. Statistical Analysis

The data are presented as the mean and standard deviation, and GraphPad Prism 6.0 was used to analyze the significance of differences based on unpaired *t* tests.

## 3. Results

### 3.1. Piceatannol Inhibited the Pore-Forming Activity of PFO

**Figure 1:**
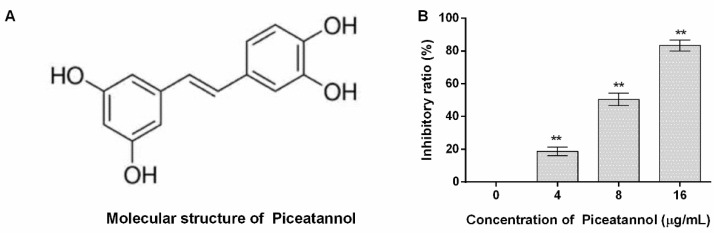
The molecular structure of piceatannol and the inhibitory effects of the compound against PFO. (**A**) The molecular structure of piceatannol. (**B**) Piceatannol inhibits the pore-forming activity of PFO. *n* = 3, ** indicates *p* ≤ 0.01.

The molecular structure of piceatannol obtained from PubChem (https://www.ncbi.nlm.nih.gov/pccompound, accessed on 23 July 2022) is shown in Figure 1A. The inhibitory ratio of piceatannol against PFO activity was defined as 0% when the concentration of piceatannol is 0 µg/mL, and the inhibitory ratios were 18.79, 50.44 and 83.34% for 4, 8 and 16 µg/mL (Figure 1B). In other words, large amounts of erythrocytes were lysed when the system was without piceatannol along with numerous hemoglobin releases. While the amount of hemoglobin sharply decreased when piceatannol was present in the reaction, and the difference was statistically significant, indicating that piceatannol inhibited the biological activity of PFO.

### 3.2. Piceatannol Bound to the Pocket between Domain One and Domain Two of PFO

**Figure 2:**
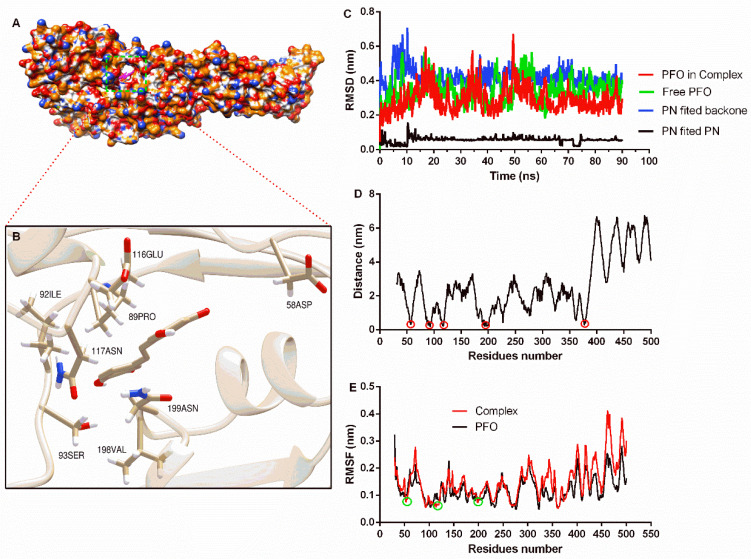
The binding mode of piceatannol and PFO and the stability of proteins, ligands and residues during binding. (**A**) The binding mode of piceatannol and PFO. (**B**) The interaction between residues and piceatannol. (**C**) The RMSD values during the molecular dynamics simulation. (**D**) The distance between residues and piceatannol. The residues in the circle show closer distance to piceatannol. (**E**) The flexibility of residues when bound with or without piceatannol. The RMSF of residues in the circle are reduced after bound with piceatannol.

The complex system obtained from molecular docking calculations showed that piceatannol was located at the junction of domain one and domain two (Figure 2A), and residues that may generate interactions are shown (Figure 2B). Then, a molecular dynamics simulation assay was performed to evaluate the reliability of the binding mode. The RMSD of the PFO backbone in the complex system showed fluctuation before 70 ns, but the trend was stable after 70 ns with a mean value of 0.27 nm. The free protein showed a similar fluctuation trend, and the RMSD mean value after 70 ns was 0.36 nm (Figure 2C), indicating that PFO possesses a stable conformation in the last 20 ns whether binding with or without the ligand. The RMSD mean value of piceatannol in the whole simulation was 0.06 nm, suggesting that it maintained a stable structure throughout the simulation. The RMSD mean value of piceatannol (fitted to the PFO backbone) was 0.42 nm and remained stable after 15 ns (Figure 2C), indicating that PFO and piceatannol maintained a stable binding mode, and the stable stage was used for the next analysis.

The distance between each residue of PFO and piceatannol was analyzed, and residues near positions 50, 100, 200 and 370 showed closer distances, indicating that these residues may interact with piceatannol (Figure 2D). The root mean square fluctuation (RMSF) can reflect the flexibility of residue. Here, the RMSF values of the residues that were closer to piceatannol were lower compared to the same residues in the free PFO, suggesting that these residues become inflexible. These results confirm the interaction between these residues and piceatannol.

### 3.3. Interaction Energy Analysis

**Figure 3:**
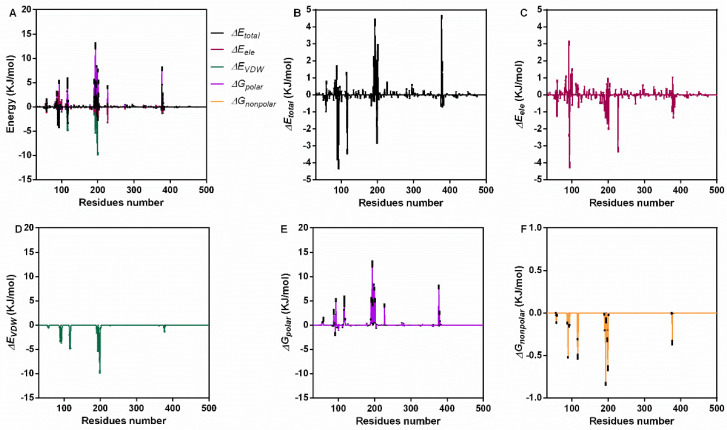
The interaction energy of piceatannol and PFO during the binding process. (**A**) The contribution of all kinds of energy. (**B**) The total energy values during binding. (**C**) The electrostatic energy. (**D**) The VDW interaction energy. (**E**,**F**) The polar and nonpolar action energies.

For further confirmation, we analyzed the interaction energy during the binding process. The total binding energy was −47.19 ± 5.63 KJ/mol, which included *ΔG_VDW_* (−134.97 ± 1.87 KJ/mol), *ΔG_ele_* (−30.13 ± 0.53 KJ/mol), *ΔG_polar_* (136.02 ± 7.88 KJ/mol) and *ΔG_nonpolar_* (−18.11 ± 0.26 KJ/mol) (Table 1). These results indicate that van der Waals forces (VDWs) are the main interaction energy, while electrostatic interaction and nonpolar solvation energy also contribute to binding, but the polar solvation energy was not conducive to binding. For a clearer understanding, the energy contribution of each residue during the stable stage was analyzed (Figure 3A). Residues near positions 100 and 200 showed higher total energy (Figure 3B), electrostatic interaction (Figure 3C), VDW energy (Figure 3D) and solvation energy (Figure 3E,F).

**Figure 4:**
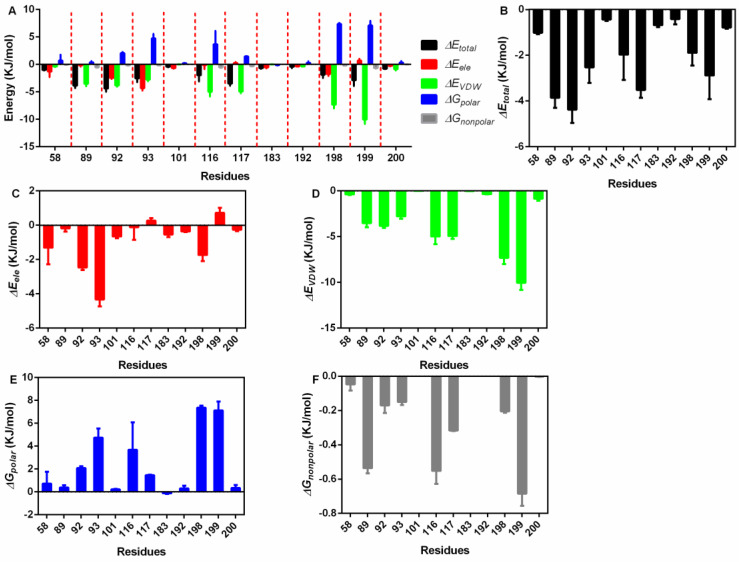
The residue energy contribution details. (**A**) The general catalogue of all kinds of energy and residues involved in the interaction. (**B**) The total energy contributions of residues that interact with piceatannol. (**C**) The electrostatic energy contributions of residues that interact with piceatannol. (**D**) The VDW interaction energy contributions of residues that interact with piceatannol. (**E**,**F**) The polar and nonpolar solvation energy contributions of residues that interact with piceatannol.

Then, the exact residues were extracted (Figure 4A), 58Asp, 89Pro, 92Ile, 93Ser, 101Arg, 116Glu, 117Asn, 183Glu, 192Ile, 198Val, 199Asn and 200Ala showed higher total binding energy among all residues (Figure 4B); 58Asp, 92Ile, 93Ser, and 198Val mainly generated electrostatic interactions with piceatannol (Figure 4C); 89Pro, 116Glu, 117Asn, and 199Asn contributed both VDW force and nonpolar interactions (Figure 4D,E), promising a relatively higher total binding energy; and 92Ile, 93Ser, 116Glu, 198Val, and 199Asn generated polar interactions (Figure 4F).

**Figure 5:**
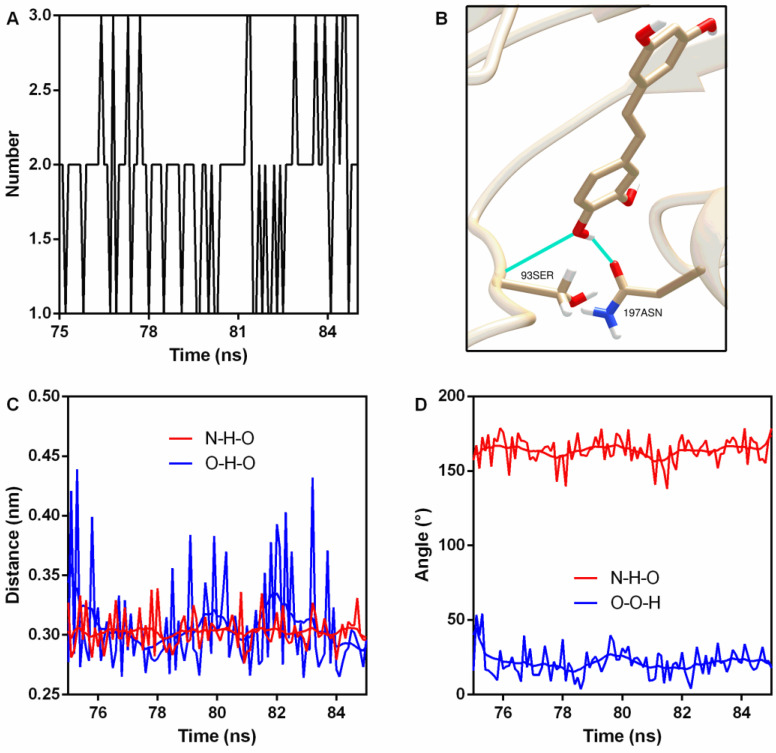
Hydrogen bonds were generated during binding. (**A**) The number of hydrogen bonds during the binding process. (**B**) The sites of hydrogen bonding between piceatannol and PFO. (**C**) The distance between the donor and receptor. (**D**) Angle analysis.

Hydrogen bonds (H-bonds) are usually generated in protein–ligand interactions. Here, two stable H-bonds were detected (Figure 5A,B); the first one was between 93Ser NH_2_ (N is the donor) and piceatannol O4 (acceptor) with an occupancy of 100% (Table 2), while the other one was between piceatannol O4 (donor), piceatannol H30, and 197Asn OD1 (acceptor), with an occupancy of 72.7% (Table 2). Analyses of the distance and angle fluctuation showed that both H-bonds were stable (Figure 5C,D and Table 2).

### 3.4. The Secondary Structure of PFO Changed after Binding with Piceatannol

**Figure 6:**
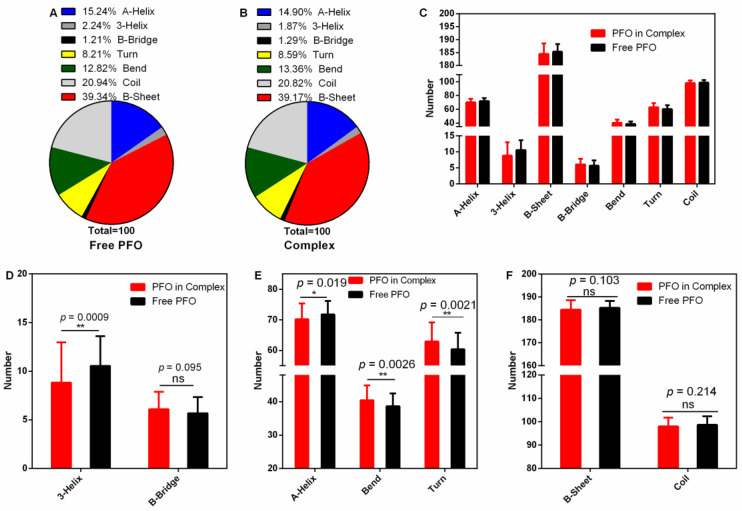
Secondary structure analysis during the stable stage. (**A**,**B**) The percentages of secondary structures in the free PFO and complex systems. (**C**) The numbers of secondary structures in the free PFO and the complex system. (**D**) The numbers of 3-helices and B-bridges in the free PFO and the complex system. (**E**) The numbers of A-helices, bends and turns in the free PFO and the complex system. (**F**) The numbers of B-sheets and coils in the free PFO and the complex system.

The secondary structure of a protein is the basis of its higher structure and affects the biological function of the protein. Here, we analyzed the changes in secondary structure when PFO was bound with or without ligand (Figure 6A–C). The numbers of 3-helices were 10.54 (percentage 2.24%) in the free protein and 8.81 (percentage 1.87%) in the complex (Figure 6A,B,D). Similarly, the numbers of A-helices were 71.77 (15.24%) in the free protein and 70.16 (14.90%) in the complex (Figure 6A,B,E). However, the numbers of bends were 37.37 in the free protein and 40.45 in the complex (Figure 6E), causing the percentage to increase from 12.82 to 13.36% (Figure 6A,B), and the numbers of turns increased from 60.39 (8.21%) to 62.93 (8.59%) (Figure 6A,B,E). All these changes were significant. However, there were no significant differences in the contents of other secondary structures (B-bridge, B-sheet and coil) (Figure 6D,F). These changes may affect the pore-forming activity of PFO.

### 3.5. Piceatannol Inhibited the Formation of PFO Oligomers

**Figure 7:**
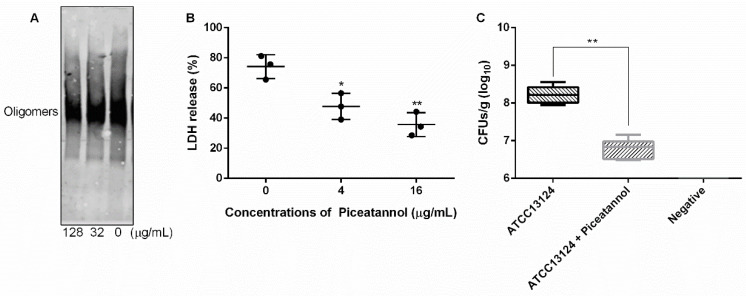
Piceatannol reduced PFO oligomer formation, cytotoxicity induced by PFO and clones of *C.* *perfringens* in mice. (**A**) The oligomer formation of PFO when treated with or without piceatannol. (**B**) Piceatannol protects cells from the damage induced by PFO. (**C**) Piceatannol reduces the clones of *C.* *perfringens* in the muscular tissue of infected mice. Three independent experiments were performed, * represents *p ≤* 0.05, ** represents *p ≤* 0.01.

As a member of a cholesterol-dependent family of toxins, the formation of oligomer is an important prerequisite for PFO to execute pore-forming activity [34]. In this work, we explored whether piceatannol affected PFO oligomer formation and found that more oligomers formed when piceatannol was not present in the system, but oligomer formation was significantly reduced when piceatannol was added to the system (Figure 7A), suggesting that piceatannol inhibited the formation of PFO oligomers in a dose-dependent manner. These results confirmed that piceatannol inhibited the biological activity of PFO by blocking the formation of its oligomers.

### 3.6. Piceatannol Alleviated the Cytotoxicity Mediated by PFO and Reduced Clones of C. perfringens in Mice

The cytotoxicity induced by *C. perfringens* mainly depends on PFO [12]. Here, we detected the effect of piceatannol on the cytotoxicity induced by PFO. The results showed that large amounts of LDH were detected in the cells treated with PFO only, and the LDH release level decreased significantly when the cells were treated with PFO and piceatannol simultaneously (Figure 7B), indicating that piceatannol inhibited the cytotoxicity of PFO and may be a candidate for the development of clinical drugs against *C. perfringens* infection.

Infected animal tests showed that the number of clones in the treatment group (mice infected with *C. perfringens* and receiving piceatannol treatment) was significantly lower than that in the infection group (mice infected with *C. perfringens* but without receiving piceatannol treatment) (Figure 7C), suggesting that piceatannol may be able to alleviate or delay the development of diseases induced by this pathogen.

## 4. Discussion

In recent decades, antibiotics have served as both antimicrobial agents and growth promoters to realize the healthy growth of livestock and poultry and to ensure the economic benefits of livestock and poultry breeding [35]. However, with the emergence and spread of bacterial resistance, the use of antibiotics has been restricted or even forbidden worldwide [36]. Thus, the livestock and poultry industries are faced with a dilemma in the reemergence of *C. perfringens* infection. Therefore, exploring and developing new anti-*C. perfringens* drugs or alternative lead compounds may be useful for combating this pathogen [37].

PFO promoting *C. perfringens* infection has been demonstrated previously, especially in the development of gas gangrene [12], which is mainly based on its pore-forming ability. In this work, we found piceatannol inhibited the pore-forming ability of PFO through a direct binding. PFO binds with cholesterol to form oligomer to execute its pore-forming ability [38]. Therefore, the inhibitory effect of piceatannol against PFO pore-forming activity may originate from its effect on oligomer formation. Here, we confirmed that piceatannol reduced the amount of PFO oligomer when compared with the group that did not receive compound treatment. PFO is the most important factor for cytotoxicity of *C. perfringens* [13]. Furthermore, PFO is essential for the survival of *C. perfringens* in host tissues; mice infected with *pfoA* gene-deficient strain could not produce the characteristic pathological damage of gas gangrene [39]; these reports confirm the important role of PFO in the generation and development of *C. perfringens* gas gangrene. In this report, we found piceatannol alleviated the cytotoxicity induced by PFO and reduced the bacterial burden in the infectious tissues.

Two other natural compounds [40,41] have been demonstrated to be able to attenuate *C. perfringens* infection by targeting PFO and ɑ toxin that is another virulence factor from this pathogen, but the action mechanisms of the proteins and the ligands were unclear. Here, we elucidated the mechanism of action between PFO and its ligand, which may provide a theoretical basis for the optimization and modification of inhibitors and may promote the rapid development of candidates into patent drugs for clinical application.

## 5. Conclusions

Piceatannol bound with PFO directly and changed part of its secondary structure, affecting the formation of its oligomer and ultimately resulting in a reduction in its pore-forming ability. Furthermore, piceatannol protected cells from the damage induced by PFO and decreased *C. perfringens* clones in the infected mice. As a foodborne natural compound, piceatannol may be a candidate for anti-*C. perfringens* infection treatment.

## Figures and Tables

**Table 1:** The binding energy between PFO and piceatannol.

Energy	Means ± Standard Deviation (KJ/mol)
*ΔG_total_*	−47.19 ± 5.63
*ΔG_VDW_*	−134.97 ± 1.87
*ΔG_ele_*	−30.13 ± 0.53
*ΔG_polar_*	136.02 ± 7.88
*ΔG_nonpolar_*	−18.11 ± 0.26

**Table 2:** The H-bonds details between PFO and piceatannol.

Donor	Hydrogen	Acceptor	Occupancy (%)	Distance (nm)	Angle (°)
93Ser N	93Ser H	PN O4	100	0.30 ± 0.014	163.91 ± 8.63
PN O4	PN H30	197Asn OD1	72.7	0.31 ± 0.040	21.20 ± 9.21

## Data Availability

The data presented in this study are available on request from the corresponding author.

## Sample Availability

Samples of the compounds are available from the authors.
